# The Law of Chance as Applied to Diagnostic Tests

**Published:** 1914-08

**Authors:** 


### The Law of Chance as Applied to Diagnostic Tests

To say that a test reacts positively in 90% of cases of a disease and in only 10% of cases not of the disease in question, affords a feeling of considerable security and, as owing to trouble and expense, the average practitioner employs the newer tests proposed only in cases in which the existence of the disease is quite probable, experience gradually leads to a firm conviction of their value.

It is not our intention, except for the sake of illustration, to mention any particular test and, from the present standpoint, it is immaterial whether the test is one depending on what is ordinarily called ''physical diagnosis'' or whether it depends on a serum reaction or some chemic manipulation of a secretion, etc. The only exception that need be mentioned is only apparent and consists in the actual demonstration of an essential point in pathology. For example, the hooking out of a portion of trichinotic muscle, the visual demonstration of a leucocythaemic state, the thorough discrimination of bacteria are really autopsies in miniature. It may be noted in passing that, here the word autopsy is more accurate than necropsy, though still somewhat objectionable. The great trouble with many of the more recent and supposedly more scientific tests is that we jump to conclusions instead of contenting ourselves with actual observations. For example, it is a truism that an excess of eosinophiles is absolutely pathognomonic of eosinophilia, but it does not necessarily mean trichinosis. We may safely say from a chemic examination there is glycosuria, even acid intoxication, but it does not certainly follow that there is diabetes; that there is albuminuria, even a shedding of renal epithelium but we can not certainly say that this or that form of nephritis is present although, in many instances, it is entirely warrantable to say that there is some form of renal degeneration. So, too, various tests applied to alimentary contents are positive so far as bleeding, stagnation, saprophytic developments, secretion, etc., are concerned but there is a wide opportunity for error if we jump to the conclusion that there is an ulcer in the restricted sense, a cancer, an organic degeneration of glands, etc.

Also, it is well to rid our minds of the assumption that, as a matter of diagnosis, any essential superiority attaches to a test requiring elaborate chemic, microscopic, radiant or other methods, or that is novel and limited in use to those possessing peculiar skill and expensive apparatus. From the practical standpoint, also, it must be remembered that a test is no more reliable than the skill of the persons using it. For example, A. L. Wolbarst (**N. Y. Med. Jour.**, Feb. 22, 1913) reports a disagreement as to the Wasserman test made by different men, all apparently well qualified, in 11 of 37 cases, 6 being different interpretations of indistinct reactions, 5 flat contradictions. No such error would be expected in regard to, let us say, the crepitant rale of pneumonia, in examinations by fairly competent practitioners. Recent reports also tend to show that the Wasserman test may be due to acid bodies in cases shown as clearly as possible by other methods, to be non-syphilitic.

If by observation of a large series of cases, any kind of a test, physical, microscopic or chemic is found to be associated with all or none of the cases of a given disease, its diagnostic value, positively or negatively, is 100%. Whether, even with allowance for inevitable human error in observation and gross blunders, any test is to this degree pathognomonic, is doubtful. If a test reacts in half of any series of cases, its diagnostic value is zero. Both of these statements require the further qualification that the positive, negative or half-and-half test is, in some way, characteristic of the disease in question. For example, if a test reacts positively in 100% of cases of a given disease and in 100% or thereabouts of various other conditions, it has no pathognomonic value although it may have a diagnostic value, as, for example, elevation of temperature as a general diagnostic of fevers. If a test reacts in 100% of a few definite diseases—if there be such a test—and nowhere else or very rarely elsewhere, it is plainly diagnostic, not of the diseases themselves, but of some common and characteristic factor. Strictly speaking, any sign approaching the pathognomonic, is a test of some characteristic feature of a disease and not of the disease itself and it is open to question whether any pathologic item subject to such a test,, is ever absolutely limited to one disease, in the clinical sense. Similar remarks apply, in a negative way, to zero percentages with tests. Likewise, the statement that a test which reacts in 50% of cases of a given diseases is of no diagnostic value, requires qualification. Theoretically, if a test reacted even in 1% of cases of a given disease and never elsewhere, it would still have some diagnostic value. Practically, a test which is indifferent with regard to any given disease, probably always is found in various other conditions.

A very important and almost neglected fallacy is applying specific diagnostic tests, and especially those recently introduced and depending on serum reactions and the like, is the a priori probability that the test will be applied, after a rather inadequate preliminary series of general investigations, to a series already selected as probably of the disease in question. Hence, if the test is at all characteristic of any factor contained in the pathologic complex of the disease studied, exaggerated ideas of its value will be confirmed.

To illustrate the influence of selection of series on results, let us assume a test which has a plus minus inaccuracy of ten per cent. So far as can be judged from various reports in literature, as to syphilis, tuberculis, cancer, etc., this is about the highest degree of efficiency of any of the newer tests, if we exclude the preliminary claims of enthusiasts and, while, there is no a priori reason for assuming that a plus error must correspond in degree to a minus error, or to any minus error at all, the fact seems to be that all tests reported do have a plus and minus error of approximately equal degree. Dropping mathematic terms, this means that there is no test beyond the early list of nearly pathognomic signs, which occurs in the great majority of cases of a given disease and which does not also occur occasionally in extrinsic cases.

Suppose, therefore, that such a test, reacting in 90% of cases of a given disease and in only 10% of extrinsic cases, is applied to a series which, by some supernatural or preternatural diagnostic power, has been determined to consist entirely of cases of the disease in question. Here, obviously, the results of the test will be correct in 90% of cases. Correspondingly, if the series were composed of cases none of which were affected with the disease in question, the exclusive diagnosis would also be correct in 90% of cases.

If the series were composed of equal numbers of individuals affected with and not affected with the disease, the results would be as follows:

| | |
|---|---|
| Persons having the disease | 500 |
| Positive reactions | 450 |
| Negative reactions | 50 |
| | |
| Persons not having the disease | 500 |
| Positive reactions | 50 |
| Negative reactions | 450 |

Here, also, the error in either the total positive or negative reactions is 10%.

Now, let us assume that the series examined consists of all the actual cases of the disease encountered and in addition, includes a number of cases in which the diagnosis is doubtful but suspicious, and that the whole series of a thousand, consists of 900 actual cases of the disease and 100 suspicious but really negative cases. This relative proportion, represents a pretty fair assortment of patients as they would occur in the practice of a fairly good diagnostician.

| | |
|---|---|
| Persons having the disease | 900 |
| Positive reactions | 810 |
| Negative reactions | 90 |
| | |
| Persons not having the disease | 100 |
| Positive reactions | 10 |
| Negative reactions | 90 |

It is obvious that, while the actual value of the test remains 90%, with a possible error of 10%, if we consider the total of 820 positive reactions, the error of ascribing the disease to persons not having it is 1 in 82, while for the total of 180 negative reactions, the wrong diagnosis will be made in half of the cases.

Suppose, on the other hand, that a physician, with the idea of being absolutely impartial and of putting his diagnoses on a reliable, objective basis, had the test applied to all of his patients and the disease in question was a fairly common one, comprising 10% (100 per mille) of his entire practice. It is obvious that the above figures would be exactly reversed. He would make a wrong diagnosis in every other positive case whiie the error would be one in 82 of negative reactions.

By preparing similar tables, consisting of 200 cases with and 800 without the disease or vice versa, or using other proportions, the error will be changed so that it becomes apparent that even with a test which has a plus minus experimental error of only 10%, the composition of the total series examined, has an influence on the net error, of surprising degree.

The fallacy of the results when we deal with rare diseases reaches startling proportions. Simply to make the illustration more definite, let us assume that we have a serum-reaction for anterior poli-myelitis or intracellularis meningitis that is 95% reliable, either positively or negatively, and that it is applied to a series of febrile cases in which the disease is suspected and in which the actual number of cases of the disease tested for, amounts to 20 per thousand. These assumptions are approximately in accordance with what may be expected, except that the assumption of an error of only 5% is, if anything, too low.

| | |
|---|---|
| Cases of poliomyelitis or meningitis.... | 20 |
| Positive reactions ..................... | 19 |
| Negative reactions .................... | 1 |
| Other fevers ........................... | 980 |
| Positive reactions ..................... | 49 |
| Negative reactions .................... | 931 |

Here we have the curious paradox that while the mistake in diagnosis of a case reacting negatively is only 1 in 932, we shall make mistakes in pronouncing the disease to be present, instead of making the clinical diagnosis of some ordinary exanthema or other febrile disturbance, in about 70% of the positive reactions.

The degree to which the fallacy depending on the actual composition of a series investigated, may reach becomes very great as we consider diseases of great rarity, in which the lack of clinical experience by the average practitioner or even by one specializing so far as possible in the study renders the discovery of a definite serum reaction especially desirable. Imagine, for example, that it were announced on good authority, that a reaction had been discovered for hydrophobia, so accurate that the chance of error in either direction, was only 1 in a thousand. Superficially, such an announcement would be acclaimed as providing a test pathognomonic within the limits of human achievement. But, if applied as a routine to the entire population, it would mean, approximately, a false diagnosis a thousand times to every correct one. Even if applied to patients actually bitten by dogs, cats and other animals, the wrong diagnoses would outnumber the correct ones many times.

It will be noted in the tables given and will be still more clearly impressed if other similar tables are made out, that the elimination of either the plus or minus error of a test, alone, would be a factor of the utmost importance. If, for example, a reaction never occurred except in a given condition, even if it occurred in only a moderate percentage of cases of that condition, we could safely submit specimens from all cases and while we could not depend except in a suggestive way upon a positive report, we would incur no danger of wrongly diagnosing, say, syphilis, cancer, or tuberculosis, and subjecting the patients from from such disease to ignominy, anxiety and the inconvenience of treatment, unnecessarily. If, on the other hand, a reaction occurred in 100% of the cases of a given disease, even if it also occasionally occurred elsewhere, the test would naturally be limited to suspicious cases and while we could not be altogether certain of its confirmatory value, the dismissal of consideration of the disease even in a small proportion of cases, would be of great service.

But, as stated, there is probably no test of the kind which has not a considerable proportion of error in either direction, unless we limit ourselves to the negative error and go back to such signs as the foetal heart beat and the crepitant rale, which are free from negative error in the sense used throughout this article, barring the lively imagination of an inexpert auscultator. And, so long as an error exists, either plus, minus or both, the absolute percentage of error as correctly stated from the standpoint of the clinical pathologist, greatly underestimates the error as it will actually occur in practice, since the composition of the series investigated influences it so largely and necessarily exaggerates it in one direction or another. It must also be realized, that with certain qualifications already mentioned, the zero of efficiency of a test starts with its 50% of incidence in a disease and reaches 100% of efficiency at either zero or 100% of incidence. Hence, unless a test occurs in close to 90% of cases of a given disease and in not over 10% of other cases or vice versa, it is, for practical purposes, of little or no value, since it may give a false sense of security on the one hand or an equally false alarm on the other, and even if the diagnosis is correct in the majority of cases in which the test is used, the exceptions are so numerous that it would be better to rely on other methods or to consider the diagnosis as merely tentative.

We apologize for the length of this article but the subject is of such vital importance—often in the most literal sense—and the misapprehension regarding it is so wide spread and apparently based on such a firm foundation, that it has seemed worth while to present the actual mathematic facts and in sufficient detail to cover the cardinal groups of cases involved.

